# Screw stent removal technique using a novel grasping device after inside plastic stent deployment for hilar biliary obstruction

**DOI:** 10.1055/a-2528-6480

**Published:** 2025-02-17

**Authors:** Takeshi Ogura, Yuki Uba, Takafumi Kanadani, Kimi Bessho, Hiroki Nishikawa

**Affiliations:** 1 13010 Endoscopy Center, Osaka Medical and Pharmaceutical University, Osaka, Japan; 2 13010 2nd Department of Internal Medicine, Osaka Medical and Pharmaceutical University, Osaka, Japan

In cases of unresectable malignant hilar biliary obstruction, uncovered self-expandable metal stent (UCSEMS) deployment using a side-by-side or stent-in-stent technique may be recommended for the purpose of prolonging stent patency, according to a consensus statement and guideline 1 2 . However, because of recent improvements in systemic chemotherapy, such as immune checkpoint inhibitors 3 , the number of reinterventions required may have increased. If a UCSEMS is deployed, reintervention may be challenging, because the UCSEMS cannot be removed.

The technique of inside plastic stent deployment has also been developed with the aim of obtaining longer stent patency. According to a recent meta-analysis 4 , inside plastic stent deployment and UCSEMS deployment have been reported to have similar efficacy. To remove an inside plastic stent, the thread must be grasped, but it may break during stent removal. Yokode et al. described a technique using rotatable grasping forceps to safely remove such stents 5 ; however, the device they used has a coiled sheath, so the removal force may be weak. Recently, a novel rotatable grasping forceps has become available (ENDO Glip; AGS Med Tech, Tokyo, Japan) ( Fig. 1 ). Unlike with conventional rotatable grasping forceps, the removal force with the novel forceps can be transmitted directly because of its noncoiled sheath. In this report, a screw stent removal technique using the novel grasping device is described ( Video 1 ).

**Fig. 1:**
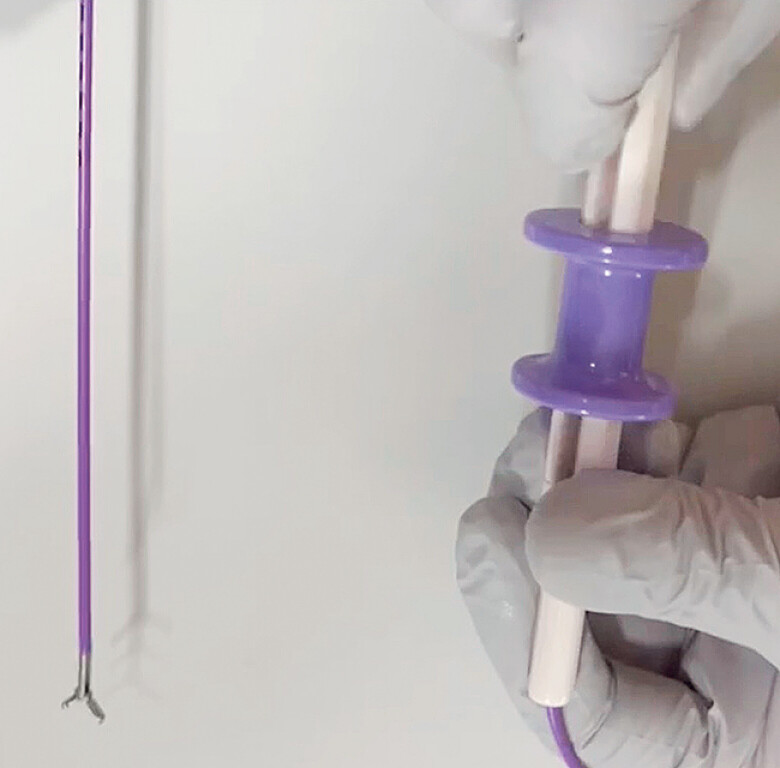
Photograph of a novel rotatable grasping forceps (ENDO Glip; AGS Med Tech, Tokyo, Japan).

A novel rotatable grasping forceps is used to remove an inside plastic stent, with the thread twisting around the forceps as the forceps is rotated, leading to successful removal of the stent without breakage of the thread.Video 1

A 71-year-old man was admitted to our hospital with recurrent biliary obstruction. He had undergone inside plastic stent deployment for cholangiocarcinoma 6 months previously. Therefore, reintervention under endoscopic retrograde cholangiopancreatography (ERCP) guidance was attempted. The duodenoscope was first inserted into the second part of the duodenum, where the thread of the inside plastic stent was identified ( Fig. 2 **a** ). After a 0.025-inch guidewire had been placed into the biliary tract, the thread was grasped using the novel rotatable grasping forceps. The grasping forceps was then rotated, with the thread twisting around the forceps ( Fig. 2 **b** ). This allowed the inside plastic stent to be successfully removed, without the thread breaking ( Fig. 3 **a** ). Finally, a new inside plastic stent was deployed ( Fig. 3 **b** ).

**Fig. 2:**
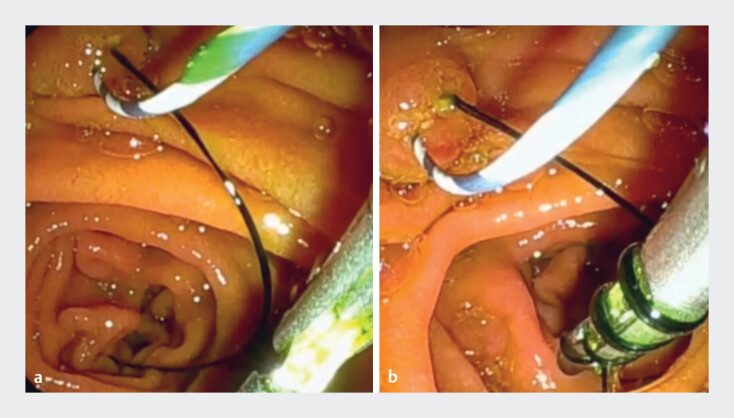
Endoscopic views showing: **a** visualization of the thread of the inside plastic stent; **b** the thread being grasped with the novel rotatable grasping forceps, which is then rotated, with the thread gradually twisting around the forceps.

**Fig. 3:**
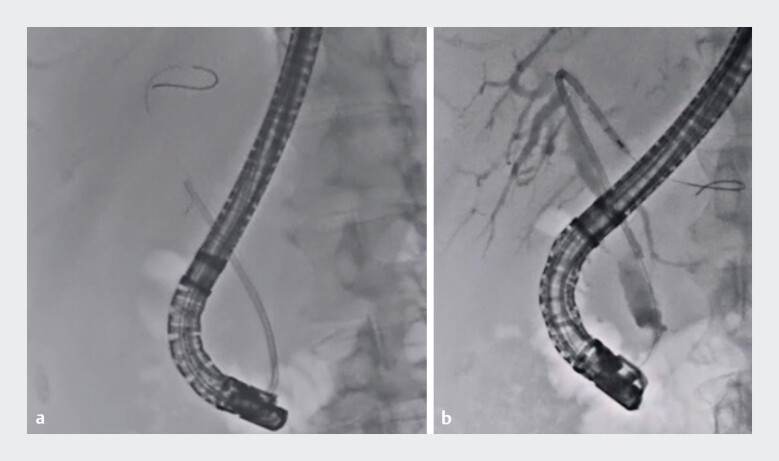
Fluoroscopic images showing: **a** the inside plastic stent being successfully removed without the thread breaking; **b** an inside plastic stent being deployed.

In conclusion, a technique using the novel rotatable grasping forceps might be useful for the removal of inside plastic stents.

Endoscopy_UCTN_Code_TTT_1AR_2AZ

## Notes

Endoscopy E-Videos https://eref.thieme.de/e-videos
*E-Videos* is an open access online section of the journal *Endoscopy* , reporting on interesting cases and new techniques in gastroenterological endoscopy. All papers include a high-quality video and are published with a Creative Commons CC-BY license. Endoscopy E-Videos qualify for HINARI discounts and waivers and eligibility is automatically checked during the submission process. We grant 100% waivers to articles whose corresponding authors are based in Group A countries and 50% waivers to those who are based in Group B countries as classified by Research4Life (see: https://www.research4life.org/access/eligibility/ ). This section has its own submission website at https://mc.manuscriptcentral.com/e-videos .
